# Anticoagulation Strategies in Venovenous Hemodialysis in Critically Ill Patients: A Five-Year Evaluation in a Surgical Intensive Care Unit

**DOI:** 10.1155/2014/808320

**Published:** 2014-12-09

**Authors:** Christoph Sponholz, Ole Bayer, Björn Kabisch, Karin Wurm, Katharina Ebert, Michael Bauer, Andreas Kortgen

**Affiliations:** ^1^Department of Anaesthesiology and Critical Care Medicine, Jena University Hospital, Erlanger Allee 101, 07747 Jena, Germany; ^2^Center for Sepsis Control and Care (CSCC), Integrated Research and Treatment Center, Jena University Hospital, 07747 Jena, Germany

## Abstract

Renal failure is a common complication among critically ill patients. Timing, dosage, and mode of renal replacement (RRT) are under debate, but also anticoagulation strategies and vascular access interfere with dialysis success. We present a retrospective, five-year evaluation of patients requiring RRT on a multidisciplinary 50-bed surgical intensive care unit of a university hospital with special regard to anticoagulation strategies and vascular access. Anticoagulation was preferably performed with unfractionated heparin or regional citrate application (RAC). Bleeding and suspected HIT-II were most common causes for RAC. In CVVHD mode filter life span was significantly longer under RAC compared to heparin or other anticoagulation strategies (*P* = 0.001). Femoral vascular access was associated with reduced filter life span (*P* = 0.012), especially under heparin anticoagulation (*P* = 0.015). Patients on RAC had higher rates of metabolic alkalosis (*P* = 0.001), required more transfusions (*P* = 0.045), and showed higher illness severity measured by SOFA scores (*P* = 0.001). RRT with unfractionated heparin represented the most common anticoagulation strategy in this study population. However, patients with bleeding risk and severe organ dysfunction were more likely placed on RAC. Citrate provided longer filter life spans regardless of vascular access site. Attention has to be paid to metabolic disturbances.

## 1. Introduction

Acute renal failure is a common complication among critically ill patients in the intensive care setting [1], increasing mortality rates up to 40–60% [2]. Renal replacement therapy (RRT) provides a therapeutic option, either as bridge until renal function recovery or as terminal therapy in loss of organ function [1]. Timing, dosage, and mode of RRT in critically ill patients are currently under debate [3]. To avoid clotting of the hemodialysis circuit anticoagulation is necessary. In the last decades, different strategies were applied for clinical application [4]. Systemic use of either unfractionated or low molecular weight heparin or regional anticoagulation with citrate (RAC) represents the anticoagulation strategies with highest acceptance within intensive care units (ICU) [5]. Although heparin anticoagulation is cheap and easy to manage when using the activated clotting time (ACT), it may enhance bleeding, particularly in postoperative patients. Moreover, it poses a risk for developing heparin induced thrombocytopenia type II (HIT-II) and leads to life-threatening complications in patients with HIT-II [6]. Due to its regional application, citrate may reduce the risk for bleeding complications. In addition, it does not induce HIT-II but has the potential to accumulate and thus may lead to metabolic as well as electrolyte disturbances. The pros and cons between both anticoagulation strategies in clinical application are therefore still under debate, resulting in conflicting results in the literature [7, 8].

This retrospective analysis aims to enlarge the knowledge about RRT in surgical critically ill patients, with special focus on anticoagulation strategies, hemodialysis filter lifetime, adverse events in clinical application, and vascular access properties.

## 2. Materials and Methods

### 2.1. Study Design

We present a retrospective, single-center evaluation of patients receiving RRT caused by acute or chronic renal dysfunction in a multidisciplinary surgical ICU, between January 2007 and December 2012. The study was approved by the local ethics committee. Need for informed consent was waived due to the anonymous and observational nature of the study.

### 2.2. Patient Recruitment

Patient data management system (COPRA, V.5.24.338, COPRA System GmbH, Sasbachwalden, Germany) was scanned for patients with need for RRT.

### 2.3. Characteristics of Hemodialysis

From 2007 to 2010 RRT was performed in continuous venovenous hemodialysis mode (CVVHD) with an aimed dialysis dose ranging from 25 to 35 mL/kg bodyweight/h. Standard anticoagulation was maintained with unfractionated heparin, adjusted to an ACT between 140 and 180 seconds. In case of active bleeding, HIT-II, or other contraindications against heparin, CVVHD was performed either with RAC, argatroban or without any anticoagulation. Dialysate fluid contained 140 mmol/L Na^+^, 2.0 mmol/L K^+^, 1.5 mmol/L Ca^++^, 0.5 mmol/L Mg^++^, 111 mmol/L Cl^−^, 35 mmol/L HCO_3_^−^, and 1.0 g/L glucose (MultiBic dialysate, Fresenius Medical Care, Bad Homburg, Germany) when using heparin, argatroban, or no anticoagulation and 133 mmol/L Na^+^, 2.0 mmol/L K^+^, 0 mmol/L Ca^++^, 0.75 mmol/L Mg^++^, 118.5 mmol/L Cl^−^, 20 mmol/L HCO_3_^−^, and 1.0 g/L glucose (Ci-Ca dialysate K2, Fresenius Medical Care, Bad Homburg, Germany) when using RAC. Citrate (4% Sodium Citrate, Fresenius Kabi, Bad Homburg, Germany) and calcium (1 N Calcium-Chloride solution, Serumwerk Bernburg AG, Bernburg, Germany) flow rates for CVVHD with RAC were predefined according to blood or dialysate flow rates, respectively (usually 120 mL/min blood flow resulting in 5 mmol/L citrate dose and 2000 mL/h dialysate flow resulting in 1.7 mmol/L calcium dose). If necessary, citrate and calcium flow rates were manually adjusted to postfilter ionized calcium levels of 0.25–0.35 mmol/L and systemic ionized calcium levels of 1.07–1.38 mmol/L by increasing or decreasing the corresponding flow rate by 0.1 mmol/L, respectively. In argatroban-CVVHD activated partial thromboplastin time was adjusted to 40–50 seconds between 2007 and 2011 and starting in 2012 argatroban was adjusted to ecarin clotting times (target: 0.5–1.0 *μ*g/mL). Vascular access for RRT was introduced in either the jugular, subclavian, or femoral vein, respectively, using a 3-lumen dialysis catheter (Trilyse Expert, Vygon, Aachen, Germany, length: 20 cm; diameter: 12 French). Maximum duration of CVVHD was 72 h per dialysis set. As standard dialysis machines Multifiltrate (Multifiltrate, Fresenius Medical Care, Bad Homburg, Germany) or Edwards bm 11 + bm 14 were in use. Starting in 2010 heparin- and argatroban-CVVHDs were largely switched to slow extended hemodialysis (SLED) using the Genius dialysis system (Genius 90, Fresenius Medical Care, Bad Homburg, Germany). In case of RAC and/or hemodynamic instability, CVVDH remained the first choice hemodialysis mode.

### 2.4. Statistical Analysis

Continuous data are presented as median [interquartile range] and categorical data as number and percentage, unless otherwise indicated. For comparison between groups, *U*-test of Mann-Whitney or Kruskal-Wallis *H*-test was applied. Evaluation of categorical data was performed using the Chi-square test. Kaplan-Meier estimates were used to compare overall hemodialysis filter life span. A *P* value <0.05 was considered to be statistically significant. Statistical analyses were performed using SPSS 13.0 (SPSS Inc., Chicago, IL, USA).

## 3. Results

### 3.1. Patient Characteristics

Within the study period, *n* = 1621 patients were placed on RRT, resulting in 10643 dialysis cycles. The majority of patients requiring hemodialysis were men and showed high APACHE-II and SAPS-II scores on ICU admission. Most patients in whom hemodialysis was deemed necessary had undergone cardiac or major abdominal surgery. 8.8% of patients required hemodialysis within the first 24 hours after ICU admission. After initiation of hemodialysis, median duration on RRT on the ICU was 3 [1–8] days, with a minimum of 1 and a maximum of 91 days. For more detailed patient characteristics, see Table 1.

### 3.2. Anticoagulation Strategies, Dialysis Performance, and Filter Lifetimes

**Figure 1:**
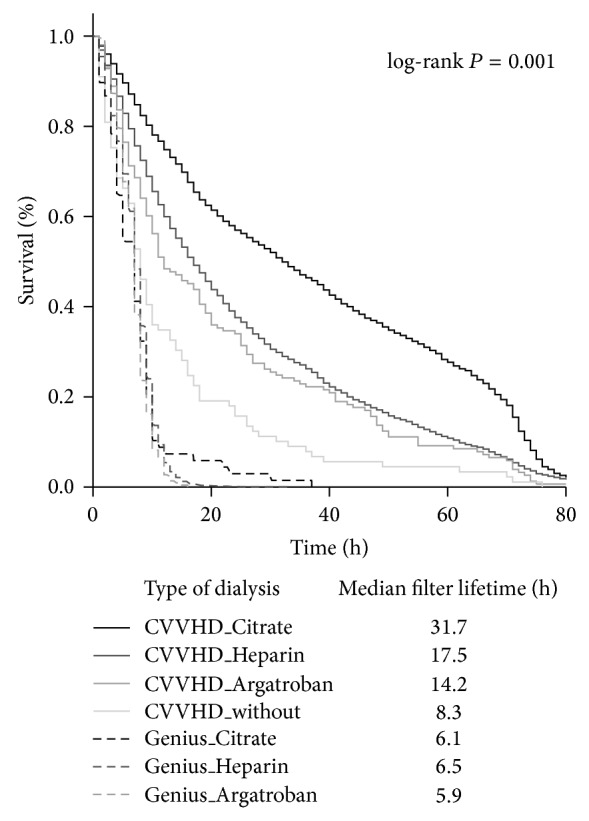
Kaplan-Meier estimates for filter survival during renal replacement therapy dependent on dialysis mode and anticoagulation strategy.

Among the *n* = 10643 performed dialysis cycles, 67% were maintained with unfractionated heparin and 28.5% with RAC, while RRTs with argatroban or without use of any anticoagulation were less frequent (3.6% and 0.8%, resp.) (Table 2). In case of CVVHD anticoagulation strategy resulted in significant different filter life spans; that is, RAC resulted in a median filter lifetime of 32 hours, while heparin anticoagulation led to a median filter lifetime of 18 hours, argatroban to 14 hours, and abandonment of any anticoagulation to 8 hours, respectively (log-rank test *P* = 0.001; see Figure 1). A filter life span of more than 24 hours was more likely to occur in patients on RAC compared to other anticoagulation strategies (*P* = 0.001; see Table 3). In contrast, no differences of filter lifetimes with respect to the anticoagulation strategy could be detected when SLED was performed. This is most likely due to the different dialysis mode with overall shorter treatment periods. Therefore, SLED was excluded from further analyses. Furthermore, CRRT without use of any anticoagulation and with use of argatroban, respectively, were performed in rather few cases, resulting in limited data. These anticoagulation strategies were also excluded from further analyses.

Hemodialysis dose in CVVHD (25.6 [22.22–30.77] mL/kg BW/h in heparin versus 25.4 [22.22–30.77] mL/kg BW/h in citrate anticoagulation) and blood flow rates (120 [100–120] mL/min in heparin versus 120 [100–120] mL/min in citrate anticoagulation) showed no clinically relevant differences between the anticoagulation strategies.

Postoperative or illness related bleeding, recurrent filter clotting under heparin anticoagulation, and proven HIT-II were the most common reasons for placement on citrate anticoagulation. However, in almost 15% of RAC, reasons for citrate anticoagulation could not be determined retrospectively (see Table 4).

### 3.3. Dialysis Access

**Figure 2:**
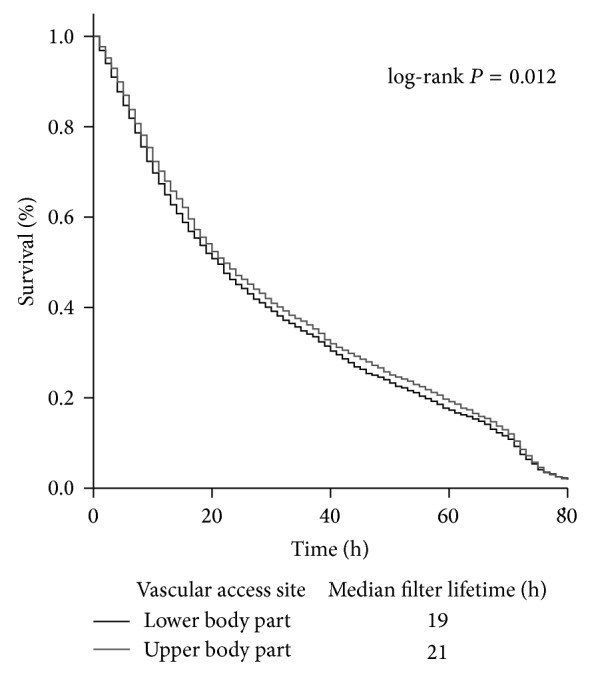
Kaplan-Meier estimates for filter survival during renal replacement therapy dependent on vascular access site.

Dialysis catheters (D-line) were mainly placed via the femoral vein (*n* = 4604 (43.3%)), followed by the right or left sided jugular vein, respectively (Table 2). Placement in the upper (jugular or subclavian vein) body part often resulted in a longer median filter lifetime (21 hours [9.0–49.0]) compared to placement in the femoral vein (19 hours [8.0–46.0], *P* = 0.012 (log-rank test, Figure 2)). With regard to anticoagulation strategy, CVVHD with heparin resulted in shorter filter life spans when the D-line was placed in the lower body site compared to upper side placement, while on the other hand RAC resulted in similar filter lifetimes regardless of D-line placement sites (Table 5).

### 3.4. Side Effects and Adverse Events during RRT with Heparin or RAC

To prevent clotting of the hemodialysis circuit heparin dosage was adjusted to an ACT between 140 and 180 seconds according to the standard operating procedure. Median ACT in all heparin RRT cycles was 166 [150.5–182.5] seconds. The aimed ACT range of 140–180 seconds was achieved in 60.1% of all measurements on heparin dialysis. ACT levels were above the aimed range in 27.8% and below in 12%.

In RAC postfilter ionized calcium levels were adjusted to a final concentration of 0.25–0.35 mmol/L and resulted in a median concentration of 0.30 [0.270–0.320] mmol/L. The intended range was achieved in 83.5% of all measurements; lower levels were measured in 8.7% and elevated levels in 7.8%. Median systemic ionized calcium levels were 1.16 [1.095–1.220] mmol/L, with 79.7% within the target concentration of 1.07–1.38 mmol/L. Elevated systemic ionized calcium levels were found in 3.2% of all cases and levels below the normal range in 17.1%.

Regarding metabolic stability, patients on heparin dialysis were more likely to develop metabolic acidosis (pH values < 7.35 and BE < −2.5 mmol/L) compared to patients on RAC, where metabolic alkalosis (pH > 7.45 and BE > +2.5 mmol/L) occurred more frequently. In detail, patients on RAC-CVVHD displayed a median pH of 7.36 [7.309–7.413], median standard bicarbonate levels of 25.0 [21.90–28.09] mmol/L, and median base excess of +1.4 [−2.39–+4.90] mmol/L. On heparin anticoagulation median pH levels were 7.35 [7.301–7.396], with a standard bicarbonate of 24.2 [21.65–25.95] mmol/L and base excess values of +0.5 [−2.55–2.50] mmol/L. Patients placed on RAC received more transfusions (erythrocytes, fresh frozen plasma, and platelets) and had higher degree of organ dysfunction measured by SOFA scores. Needs for vasopressor (norepinephrine) were similar between both anticoagulation strategies (Table 6).

## 4. Discussion

In this study we retrospectively analyzed patients treated with renal replacement therapy from 2007 to 2012 in a surgical ICU of a university hospital. We compared different anticoagulation strategies. As a major result, anticoagulation with citrate resulted in longer duration of CVVHD circuits compared to other anticoagulation strategies (i.e., unfractionated heparin). Moreover, vascular access in the femoral vein resulted in shorter filter life spans in comparison to upper body access, particularly when CVVHD was performed with heparin anticoagulation. In case of RAC filter lifetimes were independent of the dialysis catheter placement site. Patients placed on heparin dialysis were more likely to develop acidosis during RRT, while patients on RAC had higher transfusion rates and more organ dysfunction.

In 2007 Uchino published a survey of renal replacement therapy for adult renal failure worldwide. In this analysis, *n* = 1006 patients were included, with almost comparable age, gender distribution, and chronic impairment of renal function on ICU admission as described in the current analysis. Unfractionated heparin was the most common anticoagulation strategy (42.9%) followed by citrate (9.9%). Interestingly about one-third of the RRTs (33.1%) were performed without any anticoagulation [9]. While this analysis was based on a mixed patient cohort, reports of RRT solely performed in surgical patients, where regional citrate anticoagulation is more likely to be administered because of postoperative bleeding complications, are mostly restricted to specific patient cohorts, for example, cardiac surgery patients [10–12].

Differences regarding filter life span in citrate or heparin based anticoagulation strategies were already in the focus before, most of them in favour of citrate anticoagulation. Nevertheless, two recent meta-analyses evaluating available randomized controlled trials regarding heparin versus citrate anticoagulation resulted in contradictory statements regarding filter life spans for the evaluated anticoagulants. While Wu et al. could not demonstrate prolonged filter life spans in RAC [7], Zhang and Hongying reported significantly longer filter life spans in RCA in comparison to heparin based protocols [8]. However, both authors objected to the inconsistency of the available clinical trials. The present study provides clinical evidence that RAC enhances filter life span in a large cohort of critically ill surgical patients.

Placement of a central venous dialysis catheter for vascular access is essential to maintain hemodialysis in critically ill patients [13]. Data about the preferable vessel for catheterization are scarce. Access via the right sided jugular vein was identified to provide the lowest complication rates regarding blood flow and infections during RRT, but also the left sided jugular, subclavian, and femoral veins may provide advantages in some patients [13, 14], particularly if the right sided jugular vein is not applicable for catheterization. In the present analysis, 43% of dialysis catheters were placed in the femoral vein. With special regard to the duration of hemodialysis circuits and choice of anticoagulation, CVVHD with heparin resulted in shorter filter life spans when placed in the femoral vein. On the other hand, RAC resulted in similar filter life spans irrespective of the dialysis placement site. Parienti et al. compared catheter dysfunction rates and dialysis performance in a randomized controlled trial in 736 critically ill patients. As a major result, placement of the dialysis catheter in either the femoral or the jugular vein did not differ with respect to risk of catheter dysfunction or dialysis performance [15]. In a subsequent analysis from the Cathedia Study Group, first and second catheterization sites, either femoral or jugular veins, were compared in a crossover design. Again, there were no differences regarding risk of catheter dysfunction or dialysis performance [16]. Reviewing the literature, studies comparing heparin or citrate anticoagulation strategies did not focus on vascular access for hemodialysis or exclusively used the femoral vein. Thus, our data indicate that duration of CVVHD hemodialysis circuits may be influenced by the dialysis catheter insertion site and the anticoagulation strategy. However, due to the retrospective design, these data should be interpreted with caution.

In the present study, the most common cause to start or switch to RAC was active or assumed patient bleeding. The incidence of bleeding complications under heparin anticoagulation during RRT is reported to range between 15 and 50% with a mortality rate of 15% [17, 18]. Because of the retrospective design, rate of active bleeding in the present analysis cannot reliably be displayed. Moreover, as heparin administration is contraindicated in active or supposed bleeding, our standard operating procedure in such events advises citrate anticoagulation. Reviewing randomized clinical studies comparing heparin and citrate anticoagulation with focus on bleeding complications, all trials describe a trend towards less bleeding complications in citrate anticoagulation [19–22], but, only in the study of Betjes and colleagues [23], this finding reached statistical significance. Regarding transfusion of red blood cells, Monchi et al. [20] and Betjes et al. [23] describe lower transfusion rates under citrate anticoagulation, while other studies report similar transfusion rates in heparin and citrate anticoagulation during RRT [21, 22, 24]. In the present analysis, patients on RAC required higher transfusion rates of erythrocytes, platelets, and fresh frozen plasma compared to heparin anticoagulation. This may in part be explained by higher bleeding rates following surgery rather than triggered by RAC in critically ill patients, as SOFA scores were also higher in this patient cohort. Moreover, our clinical approach to allocate patients with bleeding complications, active bleeding, or poor coagulation to RAC is most likely the cause for higher transfusion requirements in the RAC cohort.

The second most common reason to switch or start on citrate anticoagulation in our study population was supposed or proven HIT-II. HIT-II is an antibody mediated prothrombotic disorder, forming antibodies against platelet-factor 4 binding to heparin. These antibodies bind and activate platelets, resulting in thrombocytopenia, thrombosis, and bleeding complications [25]. During ICU stay, 30–50% of patients exhibit thrombocytopenia, but only 1% of ICU patients develop HIT-II [26]. Recently, Sakr and coworkers described prevalence and incidence among surgical ICU patients of 0.82% and 0.62%, respectively, with a higher rate in cardiac surgery patients [6]. In the present study, cardiac and major abdominal surgery patients represented the group of patients with the highest rate of supposed or proven HIT-II, without significant differences among the groups. RRT in patients with supposed or proven HIT-II must be switched to heparin-free anticoagulation strategies to avoid heparin related complications. Patients with supposed or proven HIT-II in the present analysis were therefore placed on either RAC or argatroban anticoagulation. Recurrent clotting of the heparin circuit was the third common reason to switch to citrate anticoagulation. While the exact reasons for recurrent clotting cannot be displayed in this retrospective analysis, three major aspects can be speculated from the literature: underdosing of heparin, heparin resistance [27], and insufficient vascular access [28]. As already discussed above, vascular dialysis catheter insertion sites may be one reason for recurrent filter clotting under heparin anticoagulation in our patient cohort. It can be speculated that switching to RAC after recurrent filter clotting may be a reason for some short filter lifetimes in the RAC group although the underlying problem was not sufficiently addressed.

Patients placed on citrate anticoagulation demonstrated higher frequency of metabolic alkalosis during renal replacement therapy compared to heparin anticoagulation. Metabolic alkalosis during citrate administration occurs if a high sodium citrate load is delivered and when citrate is metabolized to bicarbonate [27]. A single center analysis including 209 patients on RAC displayed a rate of 50% of metabolic alkalosis during RRT [29]. However, following prescribed protocols, metabolic disturbances during RAC could be managed by either increasing the dialysate flow or reducing the blood flow rates. The higher rate of metabolic acidosis during heparin anticoagulation in the present patient cohort is likely to represent metabolic disturbances in critically ill patients in general that are not related to heparin anticoagulation, that is, sepsis, shock, or acute renal failure.

Our analysis has some limitations, most notably perhaps of the retrospective design. However, the retrospective approach also represents a realistic view of the daily care of critically ill patients requiring hemodialysis and may therefore not be biased by focused prospective examinations with multiple exclusion criteria and special care in study patients.

## 5. Conclusion

In the present analysis, unfractionated heparin was the most common anticoagulation strategy during RRT in critically ill surgical patients. Regional citrate anticoagulation represented a feasible alternative to extend filter life span for renal replacement therapy or in the presence of contraindications against heparin use. Vascular access may alter filter life span especially in heparin based anticoagulation protocols. Particular attention has to be paid on metabolic disturbances in citrate anticoagulation.

## Figures and Tables

**Table 1:** Characteristics of patients on renal replacement therapy on ICU admission.

Age (years)	65 [53.2–72.6]
Gender, male *n* (%)	7347 (69.0)
ICU stay (days)	26 [14–45]
ICU survival, *n* (%)	6341 (59.6)
Health scoring on admission	
APACHE-II	23 [19–30]
SAPS-II	51 [39–64]
Referring department, *n* (%)	
Cardiac surgery	5500 (51.7)
Abdominal/visceral and vascular surgery	4074 (38.3)
Traumatology	389 (3.7)
Neurosurgery	225 (2.1)
Internal medicine	185 (1.7)
Other^*^	269 (2.5)
Need for RRT, *n* (%)	
On admission	932 (8.8)
After discharge	2556 (24.0)

^*^Urology, gynecology, ear, nose, and throat, and maxillofacial surgery; ICU: intensive care unit; APACHE-II: acute physiology and chronic health evaluation; SAPS-II: simplified acute physiology score; RRT: renal replacement therapy.

**Table 2:** Anticoagulation strategies and vascular access for renal replacement therapy.

Anticoagulation strategy, *n* (%)		Vascular access, *n* (%)	
CVVHD_Heparin	3593 (33.8)	Femoral vein	4604 (43.3)
Genius_Heparin	3543 (33.3)	Right sided jugular vein	2149 (20.2)
CVVHD_Citrate	2956 (27.8)	Left sided jugular vein	1663 (15.6)
Genius_Citrate	75 (0.7)	Right sided subclavian vein	959 (9.0)
CVVDH_Argatroban	156 (1.5)	Left sided subclavian vein	1098 (10.3)
Genius_Argatroban	230 (2.2)	Not documented or another vascular access	170 (1.6)
CVVHD_without anticoagulation	90 (0.8)		

**Table 3:** Duration of CVVHD filter lifetime dependent on anticoagulation strategy.

		<24 h duration	24–72 h duration	>72 h duration	Total
CVVHD_Citrate	*n* (%)	1279 (43.3)	1319 (44.6)	358 (12.1)	2956 (100)
CVVHD_Heparin	*n* (%)	2231 (62.1)	1198 (33.3)	164 (4.6)	3593 (100)
CVVHD_Argatroban	*n* (%)	103 (66.0)	48 (30.8)	5 (3.2)	156 (100)
CVVHD_without	*n* (%)	74 (82.2)	15 (16.7)	1 (1.1)	90 (100)

Total	*n* (%)	3687 (54.3)	2580 (38.0)	528 (7.8)	6795 (100)

**Table 4:** Reasons for citrate anticoagulation among the study population.

Reasons for citrate anticoagulation, *n* (%)	
Bleeding, active or assumed	1806 (61.1)
Recurrent filter clotting under heparin	295 (10.0)
HIT-II proven	273 (9.2)
HIT-II supposed	88 (3.0)
Other	56 (1.9)
Not documented	438 (14.8)

HIT-II: heparin induced thrombocytopenia type 2.

**Table 5:** Duration of hemodialysis circuit and filter life span in relation to anticoagulation strategy.

Duration of CVVHD circuit [hours]				
Anticoagulation	Placement of dialysis catheter			
	Femoral	Jugular or subclavian	Total	*P* value
Heparin	15 [7.0–36.0]	17 [8.0–38.0]	16 [7.0–37.0]	0.015
Citrate	31 [11.0–64.0]	31 [12.0–63.0]	31 [12.0–64.0]	0.672
*P* value	0.001	0.001	0.001	

Filter life span				

Anticoagulation	<24 hours	24–72 hours	>73 hours	

Heparin	2231 (62.1%)	1198 (33.3%)	164 (4.6%)	0.001
Citrate	1279 (43.3%)	1319 (44.6%)	358 (12.1%)	
Total	**3510 (53.6%)**	**2517 (38.4%)**	**522 (8.0%)**	

**Table 6:** Side effects during renal replacement therapy among the study population.

	All	CVVHD_Heparin	CVVHD_Citrate	*P* value
pH, *n* (%)				
<7.35	2105 (32.8)	1199 (34.1)	906 (31.3)	0.001
7.35–7.45	3133 (48.9)	1819 (51.7)	1314 (45.5)	
>7.45	1174 (18.3)	503 (14.3)	671 (23.2)	
Transfusion rates:				
Erythrocytes	1 [1-2]	1 [1-2]	2 [1-2]	0.045
Platelets	0 [0-1]	0 [0-0]	0 [0-1]	0.001
FFP	0 [0–4]	0 [0–3]	0 [0–4]	0.001
Dose of norepinephrine [*µ*g/kg BW/min] (median [IQR])	0.12 [0.030–0.361]	0.13 [0.029–0.378]	0.11 [0.033–0.344]	0.896
SOFA score (median [IQR])	12 [9.0–14.0]	11 [8.0–14.0]	12 [9.0–15.0]	0.001
